# FDA‐Approved Zuranolone: First Oral Treatment for Postpartum Depression, Ushering in a New Era of Hope; A Narrative Review

**DOI:** 10.1002/hsr2.70513

**Published:** 2025-03-02

**Authors:** Muhammad Haris, Sameen Mukhtar, Mubashir Mohiuddin, Suhaina Amir, Fatima Laique, Muhammad Mazhar Azam, Bibek Giri

**Affiliations:** ^1^ Department of Medicine Dow University of Health Sciences Karachi Sindh Pakistan; ^2^ Research Institute for Collaborative Development Kathmandu Bagmati Nepal

**Keywords:** antidepressant, postpartum depression (PPD), psychotherapy, zuranolone

## Abstract

**Background and Aims:**

Postpartum depression (PPD) is a significant concern globally, emerging from various factors including hormonal changes, genetic influences, and environmental stressors. Conventional treatments such as psychotherapy and antidepressants have been the standard approach, but the recent U.S. Food and Drug Administration (FDA) approval of zuranolone presents a promising alternative with rapid onset of action and potential for increased treatment accessibility. This review aims to evaluate the efficacy and safety of zuranolone in treating PPD, considering its impact on symptom relief and patient outcomes.

**Methods:**

This review examines the multifaceted nature of PPD, focusing on its epidemiology, pathophysiology, and therapeutic interventions. Relevant literature was identified through systematic searches of databases, including PubMed and Google Scholar, using keywords related to PPD, hormonal changes, genetic factors, and treatment modalities.

**Results:**

Two rigorous phase 3 trials involving female participants diagnosed with PPD demonstrated that patients treated with zuranolone exhibited significantly greater improvement in depressive symptoms compared to those receiving a placebo. The treatment effect persisted 4 weeks after the last dose of zuranolone, indicating sustained benefits. Zuranolone showed notable effectiveness in reducing PPD symptoms as early as the third day of therapy. Its ease of administration, dosage flexibility, and potential for early intervention contribute to its promise in addressing severe PPD.

**Conclusion:**

PPD poses a significant challenge globally, and zuranolone represents a historic advancement in its treatment. While conventional therapies remain vital, zuranolone offers rapid relief and increased treatment accessibility. However, concerns regarding its safety for breastfeeding women and potential side effects necessitate further research and cautious use. Despite these challenges, zuranolone offers hope for improved outcomes and enhanced quality of life for individuals affected by PPD.

## 1 Introduction

The onset of childbirth elicits a range of powerful emotional responses, spanning from joy to anxiety. Nevertheless, it can also lead to an unforeseen outcome: the emergence of depressive symptoms. Postpartum depression (PPD) represents a complex amalgamation of physiological, emotional, and behavioral changes experienced by some women in the postpartum period [1]. It is characterized as a subtype of major depression, with manifestations typically surfacing in the first few weeks post‐birth [2]. The assessment of PPD is contingent not only on the timeframe elapsed since childbirth but also on the intensity of the depressive symptoms [1].

Primary contributors to the onset of PPD often stem from social factors, predominantly involving stressful life events, childcare‐related stress, and the predictive significance of prenatal anxiety [3]. Furthermore, the presence of a prior episode of PPD [4], marital discord, and the status of being a single parent also emerge as predictive indicators for the development of PPD [5]. Regarding lifestyle, pivotal elements such as dietary patterns, physical activities, and exercise exert a substantial influence on PPD. Vitamin B6 itself contributes to it by facilitating the conversion of tryptophan to serotonin, thereby influencing mood. Disturbed sleep cycles, frequently correlated with reduced sleep duration, are associated with PPD [6].

PPD symptoms may be characterized by mild to severe symptoms that can impede one's capacity to nurture the infant and manage daily responsibilities. Indications of PPD encompass mood fluctuations, challenges in bonding with the infant, social withdrawal, alterations in appetite and sleep patterns, profound fatigue, diminished interest in formerly pleasurable activities, irritability, and contemplation of harm to the infant [2]. The profound sense of powerlessness experienced by mothers enduring hardship, especially those grappling with PPD, leads them to describe life as an overwhelming expanse of despair [7]. This crippling feeling of hopelessness, particularly in severe cases of illness, poses a significant threat to life, potentially culminating in suicidal tendencies [8]. This factor contributes to 20% of maternal deaths in the aftermath of childbirth [9].

Epidemiological investigations have unveiled increased prevalence rates of PPD across diverse global cultures [10, 11], ranging approximately from 6.5% to 20% among women [12]. However, the prevalence of PPD is thought to exceed the reported data, with nearly 50% of mothers experiencing PPD go on (or remain) undiagnosed by healthcare professionals [13]. This condition exhibits a higher occurrence, particularly among adolescent females, mothers delivering premature infants, and individuals residing in urban locales [13].

## 2 Pathophysiology of PPD

Improving understanding of the pathophysiological mechanisms that underlie PPD may facilitate the identification of women who are at risk for PPD, as well as the development of prevention and intervention methods.

A plethora of theories have been proposed to clarify the pathophysiological mechanisms underlying postnatal depression (PND). These theories include pathways related to endocrine, epigenetic, genetic, synaptic transmission, neural network, neurosteroid, stress, and inflammation [14]. Researchers suggest that a cohesive synthesis of these many theoretical models could lead to a thorough understanding [15].

Numerous studies demonstrate the critical role that reproductive hormones play in determining depressive inclinations, suggesting a neuroendocrine basis for PPD. There is ample evidence to support the idea that changes in reproductive hormones cause dysregulation, especially in vulnerable individuals [12]. PPD is characterized by a variety of pathophysiological changes in the biological and endocrine systems, such as the immune system, the hypothalamic‐pituitary‐adrenal (HPA) axis, and lactogenic hormones [12]. Interestingly, because the HPA axis regulates cortisol release in response to stress and trauma, it is crucial to the development of PPD. This is because any interference with the HPA axis blunts this response and with HPA axis dysfunction, the release of catecholamines is reduced, hence a poor response towards stress [12]. There's a noticeable spike throughout pregnancy and for up to 12 weeks after giving birth in HPA‐releasing hormones, further implicating their role in the development of PPD [12].

For those who are vulnerable, the rapid swings in reproductive hormones like progesterone and estradiol after giving birth can be a major source of stress and may even trigger the onset of depression [16]. Additionally, because they control the production of breast milk and the milk ejection reflex, oxytocin and prolactin are important factors in the pathophysiology of PPD [16]. Notably, the start of PPD and lactation failure frequently occur at the same time. Reduced oxytocin levels are commonly seen in PPD patients and in those who stop breastfeeding too soon. Moreover, there is a correlation between elevated depressive symptoms during pregnancy and after childbirth and decreased oxytocin levels during the third trimester [16].

Empirical evidence derived from the Australian twin sample [17] and family studies suggests the existence of a hereditary component in PPD [18, 19]. Genome‐wide studies have identified candidate genes and pathways linked to PPD, focusing on those associated with major depressive disorder like serotonin transporter, Tryptophan hydroxylase 2 (TPH2), Catechol‐O‐Methyltransferase, Monoamine oxidase, and Brain‐derived neurotrophic factor (BDNF) [20]. Analysis suggests the involvement of estrogen signaling and the HPA axis. One study found 44 risk variants, including a key regulator of the stress response [21]. PPD may be influenced by epigenetic factors in addition to genetic variables [22, 23, 24, 25]. Epigenetic mechanisms mostly entail changes in chromatin structure, such as methylation or histone modifications, which affect gene transcription [20]. These changes in gene expression occur independently of variations in DNA (deoxyribonucleic acid) sequences. Environmental variables are the catalyst for these epigenetic modifications in gene expression, highlighting the dynamic interplay between genetic predispositions and environmental circumstances [20].

Neuroactive steroids, also referred to as neurosteroids, are metabolic byproducts of steroid hormones that exert influences on brain functions. Specifically, allopregnanolone, derived from progesterone, is recognized for its capacity to alleviate anxiety and depression symptoms [26]. Evidence suggests that fluctuations in allopregnanolone levels are implicated in the onset of PPD [26], with reduced concentrations correlating with increased depressive symptoms in late pregnancy [27].

## 3 Current Therapeutic Agents for PPD

The primary approach to treating PPD involves a combination of psychotherapy and antidepressant drugs as the initial course of action. Many mothers experiencing PPD are cautious about using antidepressants due to concerns about medication transmission to their infants through breastfeeding or potential adverse effects [28]. Consequently, they often prefer psychosocial and psychological psychotherapy as their first‐line treatment option for mild to moderate PPD [29, 30, 31]. Seeking guidance from a psychiatrist, psychologist, or other mental health expert may prove advantageous in addressing these concerns [2]. Therapy provides an avenue to explore and resolve issues, cultivate effective coping mechanisms, tackle challenges, establish achievable objectives, and adopt a constructive approach to situations. Moreover, family or relationship therapy can offer valuable support. Common therapeutic approaches for PPD encompass cognitive‐behavioral therapy (CBT) and interpersonal psychotherapy [2]. However, the recommended approach for moderate to severe depression entails a blend of therapy and antidepressant medication rather than just psychotherapy. Selective serotonin reuptake inhibitors (SSRIs) are typically the initial preference [32]. Should SSRIs prove ineffective, transitioning to serotonin‐norepinephrine reuptake inhibitors or mirtazapine is advisable [32]. Once an optimal dosage is achieved, it is advisable to maintain treatment for a duration of 6–12 months to mitigate the risk of symptom recurrence [32].

Pharmacological advice for nursing women should address the risks associated with antidepressants, the benefits of breastfeeding, and the risk of untreated disease. For nursing women who are concerned about their exposure to medications, there is an alternative: repetitive transcranial magnetic stimulation, which is a noninvasive procedure employing magnetic waves to activate underactive nerve cells in major depression cases [33]. Typically administered five times weekly for 4–6 weeks, it targets individuals unresponsive to antidepressants and psychotherapy. While generally safe, side effects such as headaches, lightheadedness, scalp discomfort, and facial muscle twitching may occur [33]. Rare but severe complications include seizures, hearing loss without proper ear protection, and mania in bipolar disorder patients [33].

The most thoroughly studied medication for the treatment and prevention of PPD is sertraline [33]. The use of serotonin reuptake inhibitors during nursing presents little risk; hence, it is recommended to continue breastfeeding while taking medication [33]. After 12 weeks, CBT monotherapy, particularly when combined with sertraline, showed encouraging outcomes, highlighting the significance of early treatment for PPD [33].

Conventional treatments such as psychotherapy and medication may not be beneficial for individuals with severe PPD. When a patient does not improve after four rounds of drug treatment, electroconvulsive therapy (ECT) is recommended. Patients who suffer from psychotic depression, show signs of suicide or infanticidal ideation, or refuse to eat, leading to malnourishment and dehydration, respond best to ECT [34, 35]. Moreover, multiple observational investigations have proposed ECT as a safer choice for breastfeeding mothers, with reduced adverse impacts observed on both the maternal and neonatal fronts [36, 37].

For patients with severe PPD unresponsive to or declining ECT, intravenous brexanolone is recommended [12]. Brexanolone, approved by the FDA in March 2019, is the first drug indicated specifically for PPD [12]. It is an aqueous formulation of allopregnanolone, a progesterone metabolite. However, its use is limited due to restricted availability and limited clinical experience [12]. Brexanolone is administered intravenously as a continuous 60‐h infusion over approximately 2.5 days and has typically rapid favorable efficacy in clinical trials, showing rapid beneficial effects in clinical trials [38, 39].

While existing antidepressants are vital in addressing PPD, they come with notable drawbacks, such as their delayed onset of action. Conventional oral antidepressants like SSRIs, such as fluoxetine or sertraline, may require 4–8 weeks before the complete antidepressant effect is observed [40]. Additionally, individuals experiencing PPD frequently confront obstacles when seeking pharmacological interventions, particularly those requiring invasive procedures or hospital visits, which further complicates their efforts to access appropriate care. CBT offers relief by targeting negative thought patterns associated with PPD symptoms. However, the limited availability of skilled therapists may hinder new mothers from consistently attending scheduled sessions, posing additional challenges in their journey towards recovery [41].

## 4 Zurzuvae (Zuranolone): First Oral Drug to Treat PPD

The FDA has recently granted approval for Zurzuvae (Zuranolone), the first oral medication specifically designed to treat PPD [42]. This marks a significant milestone in mental health care, offering individuals suffering from PPD a novel treatment option for rapid symptom relief [42]. Zuranolone stands out as the second FDA‐approved therapy tailored specifically for PPD [40].

Zuranolone is classified within the category of drugs known as positive allosteric modulators of gamma‐aminobutyric acid type A (GABA‐A) receptors [41]. These receptors are pivotal components of the central nervous system responsible for regulating mood, anxiety, and various emotional states. Zuranolone's mechanism of action involves augmenting the activity of GABA‐A receptors [41]. These receptors function as inhibitory neurotransmitter receptors, and their stimulation results in a decrease in neuronal excitability, thereby inducing relaxation and calming effects. By serving as a positive allosteric modulator, it enhances the responsiveness of GABA‐A receptors to the neurotransmitter GABA, thereby producing anxiolytic and antidepressant effects [41].

The effectiveness of zuranolone in treating PPD was evaluated through two rigorous phase 3 trials, employing a randomized, double‐blind, placebo‐controlled, and multicenter design [43]. These trials involved female participants diagnosed with PPD as per the Diagnostic and Statistical Manual of Mental Disorders criteria for a major depressive episode, with symptoms onset either in the third trimester of pregnancy or within 4 weeks post‐delivery [43]. A total of 196 patients were randomly assigned (zuranolone, *N* = 98; placebo, *N* = 98), and 170 participants (86.7%) completed the 45‐day study period. In the first study, patients were administered 50 mg of zuranolone or a placebo once daily in the evening for a duration of 14 days [43]. Similarly, in the second study, patients received an alternative zuranolone product approximately equivalent to 40 mg of zuranolone or a placebo, also for 14 days [44]. Following the treatment period, patients in both studies were monitored for at least 4 weeks. The primary measure of efficacy in both trials was the change in depressive symptoms as assessed by the total score on the 17‐item Hamilton Depression Rating Scale (HAM‐D) on day 15.^43,44^Additionally, in the first trial, secondary endpoints included assessing alterations in HAM‐D scores on days 3, 28, and 45, as well as evaluating changes in severity based on the Clinical Global Impressions scale on Day 15 [43]. Throughout the duration of the trial, thorough monitoring and recording of adverse events were conducted. The results revealed that patients in the zuranolone arm exhibited significantly greater improvement in their symptoms compared to those in the placebo arm. Importantly, this treatment effect persisted at Day 42, which marked 4 weeks after the last dose of zuranolone [43, 44].

When opposed to injections or hospital stays, zuranolone provides an unobtrusive and less conspicuous alternative [41]. This quality may make more people who are suffering from the symptoms of PPD seek assistance. Patient compliance rates may be higher by its ease of administration and inclusion into regular activities [41]. Furthermore, its possible implementation in diverse healthcare contexts could enhance the availability of PPD treatment in both urban and rural regions. Healthcare professionals can tailor treatment regimens to the unique requirements of each patient thanks to the dosage flexibility [41]. Additionally, zuranolone facilitates early intervention for PPD and holds promise for reducing the need for hospitalization [41]. Research results also show that zuranolone was notably effective in reducing symptoms related to PPD, with benefits seen as early as the third day after therapy initiation [40]. Thus, the rapid onset of action observed with zuranolone presents potential benefits for individuals grappling with severe PPD, as prompt relief of symptoms may be particularly advantageous in this context.

Although there is optimism due to the FDA's approval of zuranolone for PPD, there are important factors and obstacles to overcome. A major concern revolves around the lack of data concerning its safety for breastfeeding women. Clinical trials instructed participants to refrain from breastfeeding while using zuranolone; thus, crucial details regarding its excretion in breast milk, potential effects on nursing infants, and influence on milk production remain unknown [45]. This limitation could discourage many women, as interrupting breastfeeding during PPD treatment can be distressing.

Moreover, the potential impact of zuranolone on activities like driving and other risky tasks is a point of concern. Patients might experience impairment without being able to accurately gauge their own condition, necessitating careful consideration [46]. To minimize risks, individuals are advised to avoid driving or operating heavy machinery for at least 12 h after taking the medication. Common side effects, including drowsiness, dizziness, and fatigue, have been reported, alongside worries about suicidal thoughts and harm to the fetus [46]. It's recommended to use effective contraception during zuranolone treatment and continue it for 1 week after administration to reduce risks [46].

## 5 Conclusion

PPD represents a significant challenge affecting many women worldwide, with its onset influenced by a variety of factors, including hormonal fluctuations, genetic predispositions, and environmental stressors. While conventional treatments like psychotherapy and antidepressants have been the mainstay, the recent FDA approval of zuranolone offers a promising alternative with rapid onset of action and potential for increased treatment accessibility. However, concerns regarding its safety for breastfeeding women and potential side effects highlight the need for further research and careful consideration of its use. Despite these challenges, zuranolone represents a historic leap forward in addressing the complex and debilitating symptoms of PPD, offering hope for improved outcomes and enhanced quality of life for affected individuals.

## Author Contributions

**Muhammad Haris:** data curation, conceptualization, investigation, writing – original draft, methodology. **Sameen Mukhtar:** conceptualization, methodology, writing – original draft, data curation, investigation. **Mubashir Mohiuddin:** writing – original draft, conceptualization, methodology, data curation, investigation. **Suhaina Amir:** conceptualization, writing – original draft, methodology, data curation. **Fatima Laique:** conceptualization, data curation, investigation, writing – original draft, methodology. **Muhammad Mazhar Azam:** writing – original draft, conceptualization, data curation, investigation, methodology. **Bibek Giri:** writing – review and editing, project administration, resources, data curation, visualization, supervision, investigation, validation.

## Conflicts of Interest

The authors declare no conflicts of interest.

## Transparency Statement

The lead author Bibek Giri affirms that this manuscript is an honest, accurate, and transparent account of the study being reported; that no important aspects of the study have been omitted; and that any discrepancies from the study as planned (and, if relevant, registered) have been explained.

## Data Availability

Data sharing is not applicable to this article as no data sets were generated or analyzed during the current study.
